# Trends in colorectal cancer incidence: a period and birth-cohort analysis in a well-defined French population

**DOI:** 10.1186/1471-2407-11-282

**Published:** 2011-06-30

**Authors:** Marion Chauvenet, Vanessa Cottet, Côme Lepage, Valérie Jooste, Jean Faivre, Anne-Marie Bouvier

**Affiliations:** 1 Registre Bourguignon des Cancers Digestifs; Inserm U866; Université de Bourgogne; CHU Dijon, F-21079 BP 87900 21079 Dijon Cedex, France

## Abstract

**Background:**

France stands among high-risk areas for colorectal cancer. Different trends in CRC incidence are reported around the world. The aim of this study was to provide temporal trends in CRC incidence over a 30-year period in a French well-defined population.

**Methods:**

Between 1976 and 2005, 17,028 new cases were registered by the Burgundy digestive cancer registry. The mean variations in age-standardized incidence rates were estimated using a Poisson regression adjusted for age for each gender and location. The cumulative risk by birth cohort of developing a cancer over the age range 0-74 years was estimated using an age-cohort model.

**Results:**

Incidence rates for right and left colon cancers increased more rapidly in males (respectively +11.7% and +10.3% on average by 5-year period) than in females (respectively +5.9% and +6.1%). It remained stable for sigmoid cancers in males (-0.1%) and decreased in females (-5.2%). It also decreased for rectal cancers both in males (-2.7%) and in females (-2.0%). The cumulative risk increased from 3.9% for males born around 1900 to 4.9% for those born around 1930 and then slightly decreased (4.5% among those born around 1950). It remained at the same level for females born around 1900 (2.7%) as for those born around 1930 (2.7%) and then slightly increased (2.9%) for those born around 1950. For right colon cancers, the cumulative risk increased strikingly in successive birth cohorts from 0.53% to 1.2% in males and 0.55% to 0.77% in females. The corresponding cumulative risks for the left colon were 0.24% and 0.42% in males and 0.14% and 0.29% in females. For sigmoid cancer, they decreased from 1.59% to 1.08% in males, and 0.88% to 0.80% in females.

**Conclusion:**

Temporal variations in incidence rates of colorectal cancers differed according to subsite, suggesting different aetiological factors and implications for diagnosis and screening strategies. Total colonoscopy must be the preferred strategy in high-risk groups or after a positive faecal occult blood test.

## Background

In France, colorectal cancer is currently the third most common cancer in men and the second in women [1]. In 2005, the number of new cases was estimated to be 37,413 for both genders with age-standardized rates of 37.7/100,000 in males and 24.5/100,000 in females [1]. Incidence in France is comparable to that found in other high-risk areas of Western Europe, North America, Australia/New Zealand, and Japan [2], whereas it remains lower in Africa and Asia. Colorectal cancers were long considered to be a homogeneous entity, but several studies suggest that their characteristics differ by anatomical subsites [3,4]. Temporal trends in incidence are related to three main factors: age, period of diagnosis and birth cohort. Study of colorectal cancers using age-period cohort models is commonly used in order to better understand the observed trends and aetiological factors connected with them [5,6]. These models also allow estimation of the cumulative risk of developing a colorectal cancer for a given birth cohort [7]. The aim of this paper is to provide updated temporal trends in colorectal cancer incidence over a 30-year period in a well-defined French population, and to specifically differentiate the influences of period of diagnosis and birth cohort.

## Methods

### Patients

The Digestive Cancer Registry records all digestive tract cancers diagnosed in two administrative districts of Burgundy, France (1,052,000 inhabitants according to the 1999 census). The French National Commission of Data Processing and Civil Liberty (CNIL) authorized the Registry to lead incidence studies. Cancer registration began in 1976 in the Côte-d'Or area and in 1982 in the Saône-et-Loire area. Information is routinely collected from pathology laboratories, university hospitals, local hospitals, private surgeons, oncologists, gastroenterologists and general practitioners, French National Health Service records to identify patients treated outside these areas, and monthly reviews of death certificates. Cases are not recorded through death certificates alone, but these are used to identify missing cases. Since information is obtained from numerous sources, we assumed that nearly all newly diagnosed cases were recorded. Registration quality and comprehensiveness is certified every 4 years by an audit of the National Institute for Health and Medical Research (INSERM) and the National Public Health Institute (InVS). A total of 17,028 cases, over a 30-year period, were registered. Cancer location was defined according to the International Classification of Diseases for Oncology [8]. The cases were grouped into 4 categories: (1) Right colon cancers (C18.0, C18.2, C18.3, C18.4) represented 28.2% of cases (2) Left colon cancers (C18.5, C18.6) represented 7.2% of cases [3]. Sigmoid cancers (C18.7) represented 25.8% of cases and (4) Rectum and rectosigmoid junction (C19.9, C20.9) cancers represented 38.1% of cases. Colon cancer with no other information (C18.9) represented 0.7% of cases.

### Data analysis

The population data used for calculating incidence rates was obtained from the Institut National de Statistiques et des Etudes Economiques (INSEE). All rates were calculated by gender and age group. For the purpose of comparison with other countries, rates were age-standardized by the direct method using the world standard population. Incidence was calculated for the entire 1976-2005 period and for each successive 5-year period. Average variations in incidence rates for the 5-year periods were estimated using a Poisson regression, adjusted for age for each gender and location. Patients were grouped according to their year of birth into birth cohorts. Each birth cohort covered 5 successive years of births. Results start with the 1900 birth cohort, which comprised the five years of births from 1898 to 1902, up to the 1950 birth cohort, which comprised the years 1948 to 1952. Time trends by birth cohort were estimated using an age-cohort model [5,6]. Poisson regression, in which the number of cancer cases are modelled as a function of age and birth cohort, was the model used [8]. For each location, the most suitable model was used to estimate by birth cohort the 0-74 year cumulative risk of developing a cancer, according to the likelihood ratio test at the 0.05 level. The cohort-specific cumulative risk of colorectal cancer is the risk of developing this disease for an individual of the cohort who had survived up to 74 years [9]. It is the sum other each year of age of the age-specific incidence rates taken from birth to age 74. Data were analyzed using Stata 10.0 ™ software [10].

## Results

### Time trends in incidence rates by period of diagnosis

Overall, the average variation in CRC incidence rates between successive five-year periods was +1.9% [95%CI: +0.6; +3.2] in males and + 0.3% [95%CI: -1.1; +2.8] in females. The trends in incidence varied according to subsite as shown in Table 1. Between successive 5-year periods, incidence rates increased significantly for right colon and left colon cancer for both genders. The average increase by 5-year period was greater in males, respectively +11.7% and +10.3%, than in females, respectively +5.9% and. +6.1%. In contrast, for sigmoid cancers, there were different trends for the two genders: incidence was stable in males and decreased in females. For rectal cancer, the incidence decreased significantly in males (-2.7% on average) and non significantly in females (-2.0% on average). Across the study period, the male-female ratio remained higher than 1 for all subsites. It tended to slightly increase for colon subsites.

**Table 1:** Age-standardized incidence rates by gender, subsite and period of diagnosis

	Incidence rates by periods of diagnosis						Average variation	
	76-80	81-85	86-90	91-95	96-00	01-05	%	[95% CI]
**Males**								
All sites	34.9	37.9	40.8	40.0	41.5	40.3	+ 1.9	[0.6; 3.2]
Right Colon	5.9	7.4	7.8	9.2	10.3	10.2	+ 11.7	[8.6; 14.8]
Left Colon	1.8	2.3	3.0	2.8	3.0	3.7	+ 10.3	[5.0; 15.8]
Sigmoid	9.6	10.1	12.0	10.2	11.0	10.6	- 0.1	[-2.5; 2.4]
Rectum	17.7	17.7	17.7	17.5	17.0	15.7	- 2.7	[-4.7; -0.8]
**Females**								
All sites	21.9	23.3	22.8	23.2	23.3	23.1	+0.3	[-1.1; 2.8]
Right Colon	5.6	5.9	6.6	6.8	7.7	7.1	+ 5.9	[3.3; 8.6]
Left Colon	1.3	1.6	2.0	1.5	1.9	2.0	+ 6.1	[0.4; 12.1]
Sigmoid	5.6	7.5	6.6	5.9	5.5	6.1	- 5.2	[-7.9; -2.5]
Rectum	9.3	8.1	7.5	8.6	8.1	7.8	-2.0	[-4.5; 0.4]

### Time trends in incidence rates by birth cohort

In males, the cumulative risk rose from 3.9% among those born around 1900 to 4.9% for those born around 1930 and then slightly decreased to 4.5% among those born around 1950. In contrast, it remained at the same level for females born around 1900 as for those born around 1930 (2.7% for both cohorts) and then slightly increased (2.9%) for females born around 1950.

The cumulative risk of developing right colon cancer over the age range 0-74 years has increased strikingly in successive birth cohorts (Table 2). It rose from 0.53% for males born around 1900 to 1.20% for those born around 1950, a 2.3-fold increase. The corresponding values in females were 0.55% and 0.77%, a 1.4-fold increase. There was a similar trend for the risk of developing left colon cancer over the age range 0-74 years. There was a 1.8-fold increase in males and a 2.1-fold increase in females. In contrast there was a decrease in the cumulative risk for sigmoid and rectal cancers between those born around 1900 and those born around 1950. For sigmoid cancers, there was a 1.5-fold decrease in males born around 1950 and a 1.1-fold decrease in females. For rectal cancers there was also a 1.5-fold decrease in the cumulative risk in males. In females, the risk decreased between those born around 1900 to those born around 1930. It then increased for those born after 1930 to reach the same level as for those born around 1900.

**Table 2:** Cumulative risk over the age range 0-74 years of developing colorectal cancer by gender and subsite according to birth cohort

	1900	1905	1910	1915	1920	1925	1930	1935	1940	1945	1950
**Males**											
All sites	3.94	4.20	4.42	4.60	4.73	4.82	4.85	4.83	4.75	4.63	4.46
Right Colon	0.53	0.62	0.72	0.82	0.92	1.01	1.09	1.15	1.19	1.21	1.20
Left Colon	0.24	0.26	0.29	0.31	0.33	0.35	0.37	0.39	0.40	0.41	0.42
Sigmoid	1.59	1.56	1.52	1.48	1.44	1.39	1.33	1.27	1.21	1.15	1.08
Rectum	2.44	2.42	2.39	2.33	2.26	2.18	2.08	1.97	1.86	1.74	1.61
**Females**											
All sites	2.68	2.67	2.66	2.66	2.67	2.68	2.70	2.73	2.76	2.80	2.85
Right Colon	0.55	0.61	0.66	0.71	0.75	0.78	0.80	0.81	0.81	0.80	0.77
Left Colon	0.14	0.15	0.15	0.17	0.18	0.19	0.21	0.22	0.24	0.27	0.29
Sigmoid	0.88	0.82	0.78	0.74	0.72	0.71	0.71	0.72	0.73	0.76	0.80
Rectum	1.09	1.04	1.00	0.97	0.95	0.94	0.95	0.96	0.98	1.01	1.05

## Discussion

Over a 30-year period, the overall incidence of colorectal cancer in France has slightly increased in males and remained stable in females. In the youngest birth cohorts the cumulative risk over the age range 0-74 years slightly decreased in males whereas it slightly increased in females. The most impressive aspect of our study was the marked increase in the incidence rates of right and left colon cancers both by time period and birth cohort. The cumulative risk of sigmoid and rectal cancers decreased in males while it remained relatively stable in females.

The Burgundy registry is the most longstanding population-based digestive cancer registry in France. The multiplicity of information sources allows us to assume that nearly all newly diagnosed cancers are recorded. Thus, it is an appropriate tool for observing time trends in incidence rates. When analyzing 30 years of incidence, it is necessary to evaluate the comparability of data over such a long period. The strength of our results lies in the fact that the registration scheme and the coding rules remained the same over the study period. Colonoscopy has progressively replaced barium enema as a diagnostic procedure. However, this was unlikely to explain time trends in incidence.

Five volumes of the publication Cancer Incidence in Five Continents provide data on time trends on colorectal cancer incidence over the period covered by this paper [11-15]. Different trends in CRC incidence can be seen around the world. In Western Europe, incidence rates were stable or slightly increased, with the exception of Norway and Spain where the increase in incidence was more pronounced. Incidence is generally on the increase in East European countries, Central and South America and in Asia. The most prominent feature was the sharp increase in incidence in Japan, which now stands among high-risk areas [16]. After an increase in incidence until the late 1980s, a decrease in incidence was reported in the USA, while it remained stable in Canada. Incidence rates were also stable in Australia and New Zealand [17].

Availability of incidence rates by subsites is more limited. Evidence exists for different time trends, in particular among high-risk areas. In the USA, stable incidence rates for right colon cancer, and declining rates for left colon (descending and sigmoid) and rectal cancer were reported over the 1992-2001 period [18]. Data covering the 1997-2006 period indicates that all CRCs are now significantly declining in the US [19]. Among available data in Europe, an increase in all colon subsites was reported in Denmark [4,20], Norway [21] and Italy [22,23], whereas in Burgundy, the increase was limited to right and left colon cancers. A recent study from Norway reveals deceleration in the rate of increase of colon cancer subsites [24]. In most high-risk areas, the incidence of rectal cancer decreased (USA, Denmark, France) or was stable (Italy, Australia), whereas it increased in Norway [24-27]. In low-risk areas of Asia, Central and South America, there was a striking increase in incidence of all colon subsites, while it was moderate for rectal cancers [11-15].

Most studies on trends in colorectal cancer incidence are limited to analysis by period of diagnosis. Trends by birth cohort bear different implications and are thus complementary. Effects of both period of diagnosis and birth cohort were found in this study, as well as in other studies [21,26,27]. However for sigmoid cancers the birth cohort effect was more marked in males and the period of diagnosis effect in females. Exposure to early-stage risk factor or protective factor with long-term latency periods over different generations will introduce a cohort effect. A period of diagnosis effect can be attributed to risk factors or protective factors involved in the late stage of carcinogenesis affecting all age groups, or to changes in screening practices. The protective role of vegetables reported in case control studies (and not in cohort studies) could be attributed to a period effect. Case control studies, concerning recent intakes, provide data on dietary factors involved in the last phase of colorectal carcinogenesis, while cohort's studies provide data on the early phase of this colorectal carcinogenesis. Dietary factors affecting the last step of the adenoma-carcinoma sequence can modify cancer risk in a relatively short period of time. An example of this period effect is given by the westernisation of diet in Japan. It became a high-risk country for colorectal cancer in about 15 years following changes in dietary behaviour [28]. A similar effect was observed in Japanese migrants to the USA [29]. There are discrepancies between case-control and cohort studies, but these are not surprising. Cohort studies collecting data on diet a long time before the appearance of the cancer are able to identify the main dietary factors related to the cohort effect, while case-control studies that take recent diet into account reveal factors related to the period effect. There is a tendency to consider that in case of discrepancies, cohort studies are more representative of the truth. As factors involved in adenoma appearance or growth differ, at least partly, from those involved in adenoma transformation, then cohort and case control studies should provide somewhat different results.

Data from the United States suggest an impact of screening on the decline in mortality and incidence from CRC. The 2010 US annual report to the Nation on the status of cancer provides the results of a simulation model for interpreting mortality trends for CRC. The results suggest that 35% of the observed mortality decline can be explained by changes in risk factors, 53% by screening and 12% by improvement in treatment [19]. However, the impact on incidence rates can only be seen after a long time lag [30]. In a cohort of patients with adenomas measuring more than 1 cm in diameter that remained in the colon, the cumulative colon cancer rate was 8% after 10 years and 24% after 20 years [31]. The marked decrease in the incidence of CRC in the US, between 1985-1995 cannot be attributed to screening, which was not widespread in the 15-20 years preceding that period. Changes in risk factors or protective factors probably played the most important role. A significant positive effect of screening on CRC mortality or incidence can be expected only during the most recent period. The decline in CRC incidence could be accelerated further by higher utilisation of screening. Trends in incidence in Europe cannot be related to screening practices because of too recent development of screening programmes. For instance Norway has not undertaken CRC screening programmes and less than 5% of colonoscopies performed are related to CRC screening [24].

The different time trends by subsite within areas, and among areas with similar overall CRC incidence, suggest that dietary factors involved in CRC carcinogenesis along the length of the colon might differ, at least partly, and that the protecting or enhancing factors may differ from one area of the world to another. For instance meat consumption is a more important risk factor in North America or Australia where consumption is higher than in Europe, and refined cereal consumption (in particular pasta and rice) are risk factors only in Latin European countries where consumption of these products is high [32]. There are also data indicating that aetiological factors may differ along the large bowel. In a case-control study performed in the Côte-d'Or area, the protective effect of high vegetable intake was restricted to the sigmoid colon. Factors that might be more linked to right colon cancer include body mass index. It has increased over the past few decades in France. The increased incidence of right colon cancer could be related to the increase in obesity.

## Conclusions

The strength of our study lies in the fact that it is a population-based study, using high-quality cancer registry data collected over a 30-year period, with time trends analysed both by period and birth cohort. Incidence rates in Burgundy are similar to those reported at the national level [1]. So we feel that our findings can be generalised to the French population overall. This study shows that the incidence increased in males and was stable in females over the 1976-2005 period. In revealing different time trends in incidence for subsites and genders, our results suggest that CRCs have, at least in part, different aetiological factors. Differences are also reported among cancer registries. Since colorectal cancer is one of the most common cancers it is important to conduct future studies that take into account environmental, genetic and molecular factors. The shift towards right colon cancers reported here has implications for the choice of colorectal cancer screening methods.

## Competing interests

The authors declare that they have no competing interests.

## Authors' contributions

MC, JF and AMB contributed to the study design, data analysis and drafting of the paper, VJ to the data analysis and drafting of the paper, VC and CL to the interpretation of the results and drafting of the paper.

All authors read and approved the final manuscript.

## Pre-publication history

The pre-publication history for this paper can be accessed here:

http://www.biomedcentral.com/1471-2407/11/282/prepub
